# Supplementary material for: Inorganic nitrogen inhibits symbiotic nitrogen fixation through blocking NRAMP2-mediated iron delivery in soybean nodules
Source: Nat Commun. 2024 Oct 17;15:8946. doi: 10.1038/s41467-024-53325-y (PMC11484902; doi:10.1038/s41467-024-53325-y)
Supplement: Supplementary file 1 — Supplementary Information [file 41467_2024_53325_MOESM1_ESM.pdf]

Supplementary information for

Inorganic nitrogen inhibits symbiotic nitrogen fixation through blocking NRAMP2-mediated iron delivery in soybean nodules.

This PDF file includes:

Supplementary figures S1 to S17

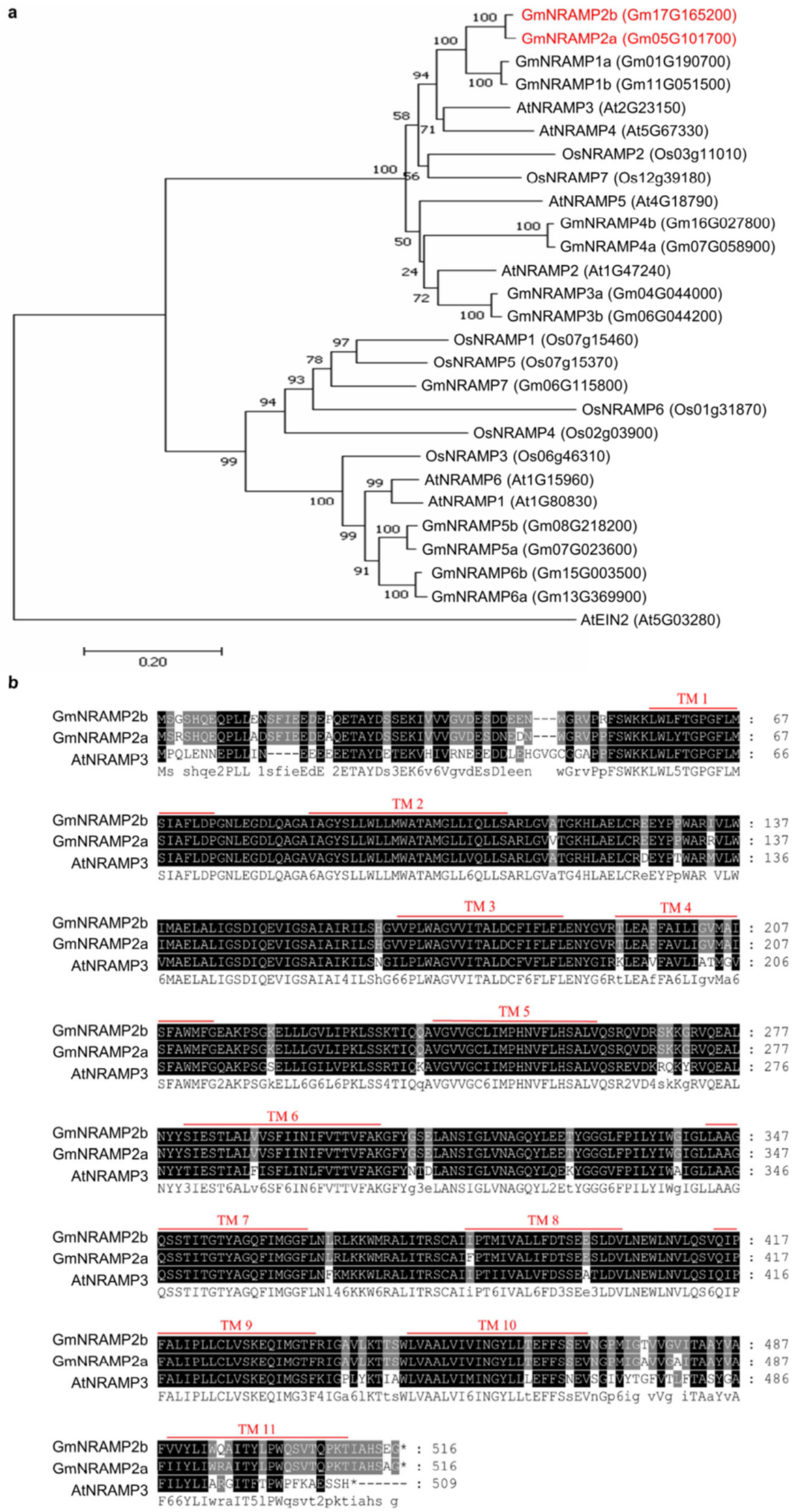

Supplementary Fig. 1. Protein similarity analysis of GmNRAMP2a&2b.

(**a**) Phylogenetic tree of NRAMP family members in soybean, rice and Arabidopsis. Protein sequences were obtained from Phytozome. The phylogenetic tree was constructed with MEGA7 using the Neighbor-Joining method and 2000 bootstrap replicates. The evolutionary distances were computed using the Poisson correction method and are in the units of the number of amino acid substitutions per site. Gm, soybean; Os, rice; At, Arabidopsis.

(**b**) Alignment of GmRNAMP2a&2b and AtNRAMP3. Protein sequences were analyzed by MEGA7 and diagramed by Gene Doc Win 2.7. Amino acid sequences identical in all three are highlighted in black, and those with two identical sequences are highlighted in grey. The transmembrane domains (TM) are predicted with TMHMM (http://www.cbs.dtu.dk/services/TMHMM/) and indicated with red lines. Gm, soybean; At, Arabidopsis.

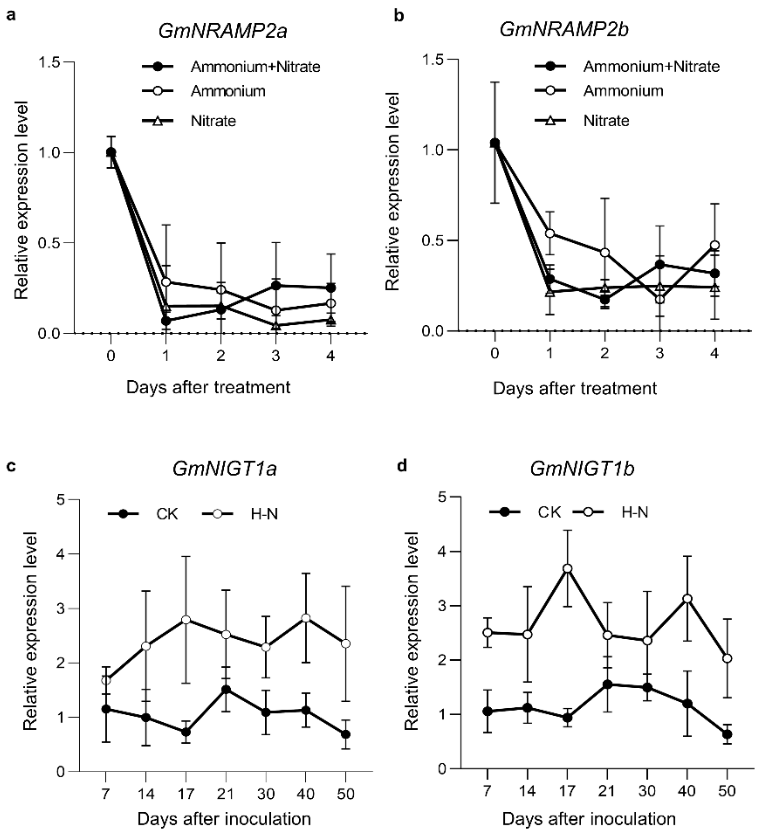

Supplementary Fig. 2. Gene expression pattern of *GmNRAMP2a&2b* and *GmNIGT1a&1b*.

(**a-b**) Expression of *GmNRAMP2a&2b* response to different nitrogen source in nodules. Nodules at 21dpi were treated with 10 mM ammonium, nitrate or a combination of both for 1, 2, 3 or 4 days.

(**c-d**) Time-dependent expression of *GmNIGT1a&1b* in nodules. 4-d-old seedlings were inoculated with rhizobia and cultured in low-N solution for different days. At each time point, the seedlings are split into two groups: one group is subject to a high-nitrogen (H-N) treatment for one day, while the other group remains untreated and serves as the control (CK). Relative expression levels were determined by real-time RT-PCR. *EF-1α* was used as an internal standard. Data are means ± SD. n = 3-5 (**a-d**) biologically independent replicates.

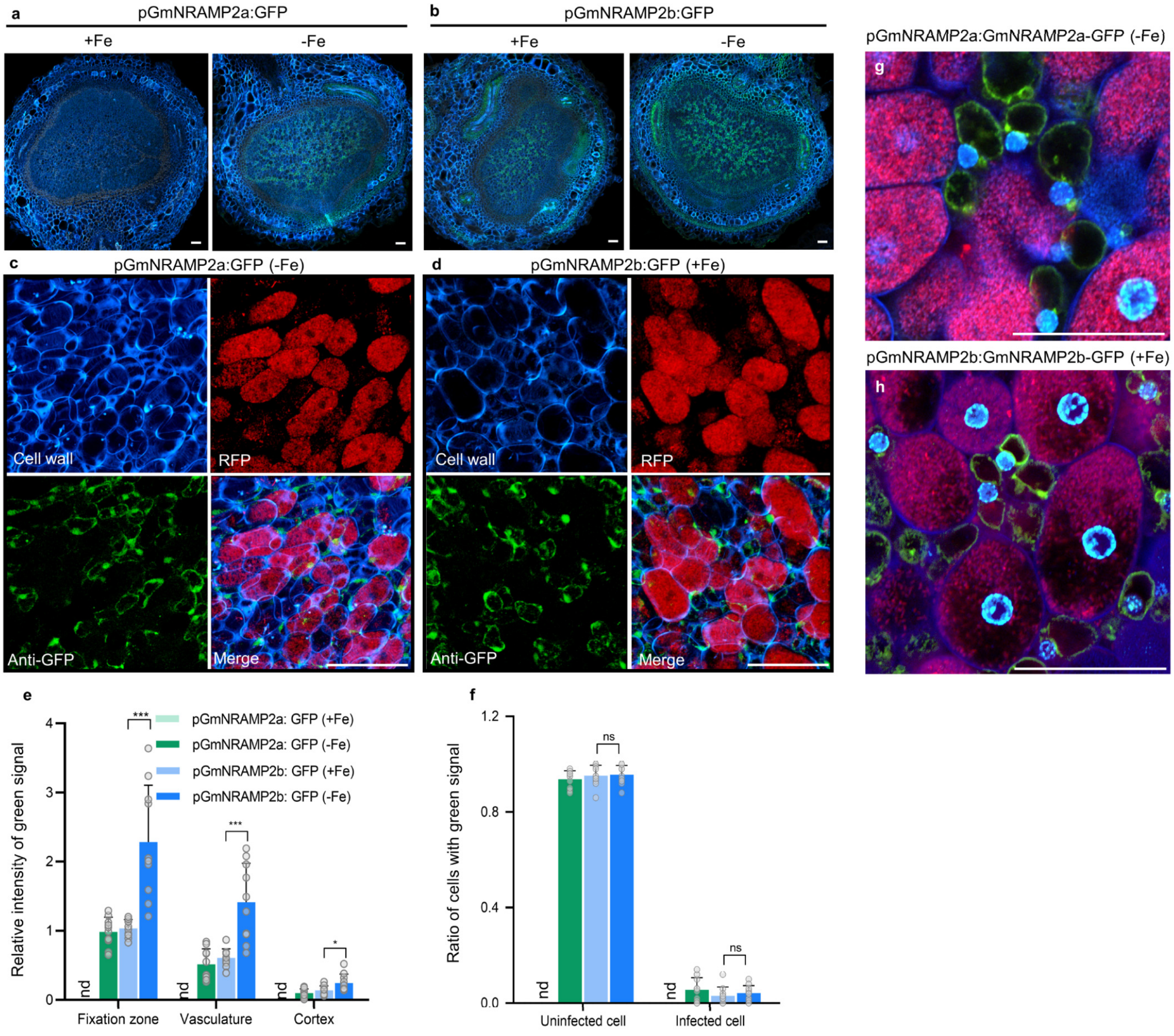

Supplementary Fig. 3. GmNRAMP2a&2b are located at the tonoplast of uninfected cells in nodules at 30 dpi.

(**a**-**d**) Immunostaining of *pGmNRAMP2a: GFP* (**a**, **c**) and *pGmNRAMP2b: GFP* (**b**, **d**) transgenic nodules. Magnified images in fixation zone are shown in (**c**, **d**), with individual channels and overlay (merge). Plants with transgenic hairy roots and nodules at 23 dpi were transplanted to Fe-free (-Fe) or Fe-sufficient (+Fe) solutions for 7 d. (**e**) Relative intensity of green signal from (**a**, **b**). (**f**) Ratio of cells with green signal in (**c**-**d**). nd means non-detected values. (**g**-**h**) Immunostaining of transgenic nodules expressing *pGmNRAMP2a/2b:GmNRAMP2a/2b-GFP*. Transgenic nodules carrying *pGmNRAMP2a/2b:GmNRAMP2a/2b-GFP* at 23 dpi were transplanted to Fe-free (-Fe) or Fe-sufficient (+Fe) solutions for 7 d. Five independent transgenic lines were investigated and consistent results were obtained, with one representative image presented in (**a**-**d**, **g**-**h**). Green shows signals from anti-GFP, and blue shows signals from cell wall. Red shows RFP-tagged rhizobia. Scale bars, 50 µm. Data are means + SD. n = 10 (**e**-**f**) replicates from independent nodules. Asterisks in (**e**-**f**) indicate significant differences compared with +Fe: $0.01 < {*}P \leq 0.05$, $0.001 < {**}P \leq 0.01$, $***P \leq 0.001$ by Student's *t*-test, two-tailed *t*-test.

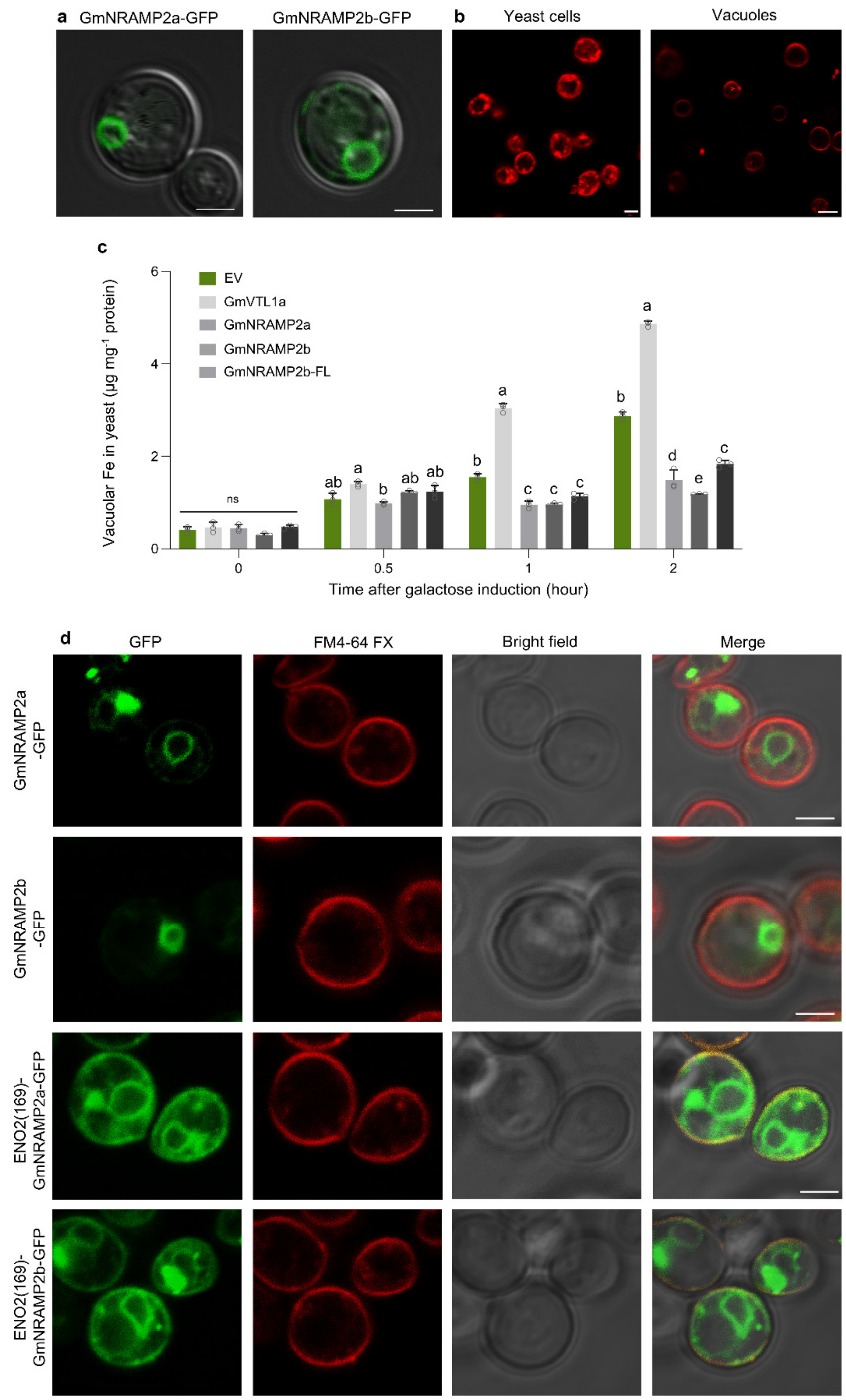

Supplementary Fig. 4. Subcellular localization and complementation test in yeast.

(**a**) Vacuolar targeting of GmNRAMP2a&2b in yeast. BY4741 strain was transformed with *GmNRAMP2a-GFP* or *GmNRAMP2b-GFP*, and fluorescence was observed with a confocal microscope. Scale bars, 2 μm. (**b**) Determination of the integrity of vacuolar fraction. Yeast cells were stained with 30 μM FM4-64 FX for 1 hour before vacuole isolation. Both yeast cells and isolated vacuoles were photographed. Scale bars, 2 μm. (**c**) Fe content in isolated yeast vacuoles. BY4741 strains were transformed with empty vector (EV), *GmVTL1a*, *GmNRAMP2a*, *2b* or *GmNRAMP2b-full length (FL)*. Yeast vacuoles were isolated by ficoll gradient centrifugation before incubation with galactose for 0, 0.5, 1 or 2 h, and then used for Fe determination by ICP-MS. The different letters in each time point indicate significant differences ($P \leq 0.05$) in multiple comparisons tests following Tukey tests. (**d**) Subcellular localization of ENO2(169)-fused GmNRAMP2a&2b in yeast. BY4741 strain was transformed with *GmNRAMP2a/2b-GFP* or *ENO2(169)-GmNRAMP2a/2b-GFP*, and fluorescence was observed with a confocal microscope before stained with FM4-64 FX for 1 min. Scale bars, 2 μm.

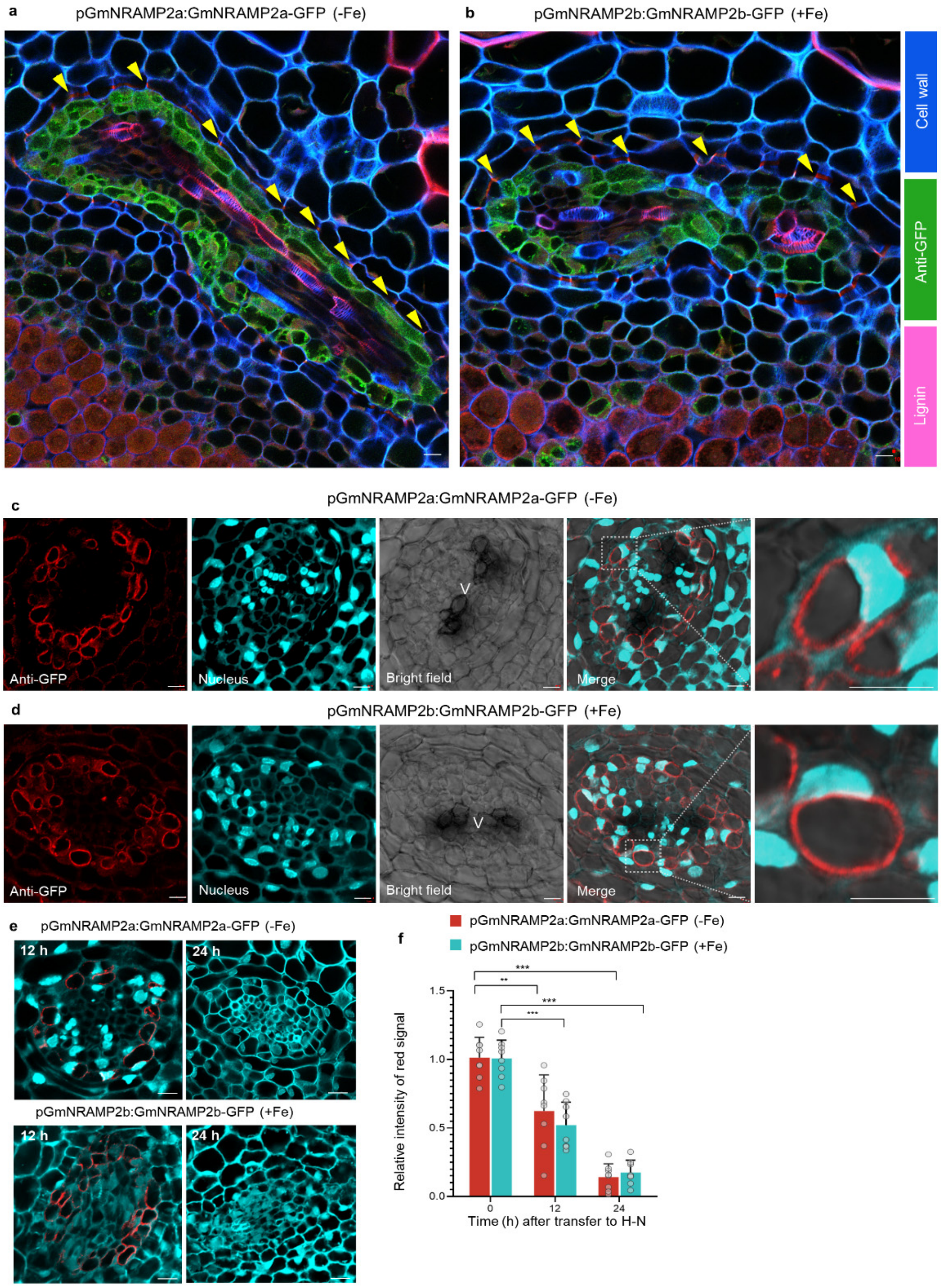

Supplementary Fig. 5. Localization of GmNRAMP2a&2b in nodule vasculature.

(**a**-**b**) Tissue-localization of GmNRAMP2a (**a**) and GmNRAMP2b (**b**). Plants with transgenic hairy roots and nodules carrying *pGmNRAMP2a/2b:GmNRAMP2a/2b-GFP* at 10 dpi were transplanted to Fe-free (-Fe) or Fe-sufficient (+Fe) solutions for 7 d, and then sampled for immunostaining. Yellow arrows indicate the endodermis layer stained with Fuchsin basic. Green shows signals from anti-GFP, and blue shows signals from cell wall.

(**c**-**d**) Subcellular localization of GmNramp2a (**c**) and GmNRAMP2b (**d**). Cyan shows signals from nucleus stained with DAPI, and red shows signals from anti-GFP. White dot areas are magnified at rightmost panels.

(**e**-**f**) Protein abundance response to N. Cyan shows signals from cell wall and nucleus. Red shows anti-GFP signals. Transgenic nodules carrying *pGmNRAMP2a/2b:GmNRAMP2a/2b-GFP* at 10 dpi from hairy roots were transplanted to Fe-free (-Fe) or Fe-sufficient (+Fe) solutions for 7 d, and then treated with high-N (H-N) for 0, 12 or 24 h. Five independent transgenic lines were investigated and consistent results were obtained, with one representative image presented in (**a-e**). Scale bars, 10 μm (**a-e**). Data are means + SD. n = 8 (**f**) replicates from independent nodules. Asterisks in (**f**) indicate significant differences compared with 0 h: $0.01 < {*P} \leq 0.05$, $0.001 < {**P} \leq 0.01$, $***P \leq 0.001$ by Student's *t*-test, two-tailed *t*-test.

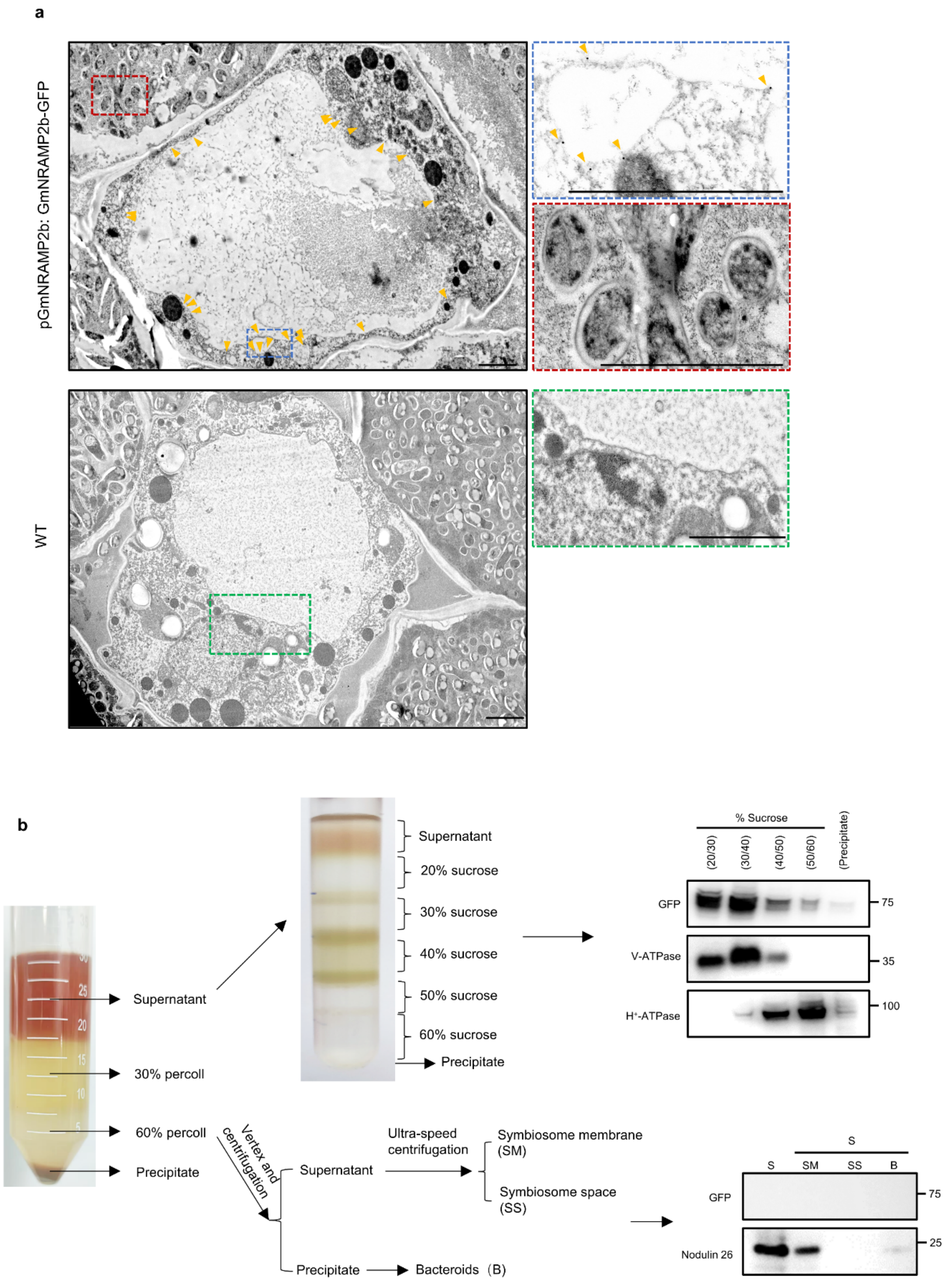

Supplementary Fig. 6. Subcellular localization of GmNRAMP2b determined by immunoelectron microscopy and western blot.

(**a**) Subcellular localization of GmNRAMP2b determined by immunoelectron microscopy. Transgenic nodules carrying *pGmNRAMP2b:GmNRAMP2b-GFP* at 21 dpi were sampled for immunostaining. The images inside the colored boxes are enlarged and displayed on the right. The yellow triangle arrows indicate the location of the immunogold. Scale bars, 2 μm.

(**b**) Subcellular localization of GmNRAMP2b determined by western blot. Transgenic nodules carrying *pGmNRAMP2b:GmNRAMP2b-GFP* at 21 dpi were sampled for immunoblot assay. The separation process of the plasma membrane (PM), tonoplast, and symbiosome membrane (SM) in nodules is shown on the left. V-ATPase is a tonoplast marker; H^{+}-ATPase is a PM marker; Nodulin 26 is a SM marker.

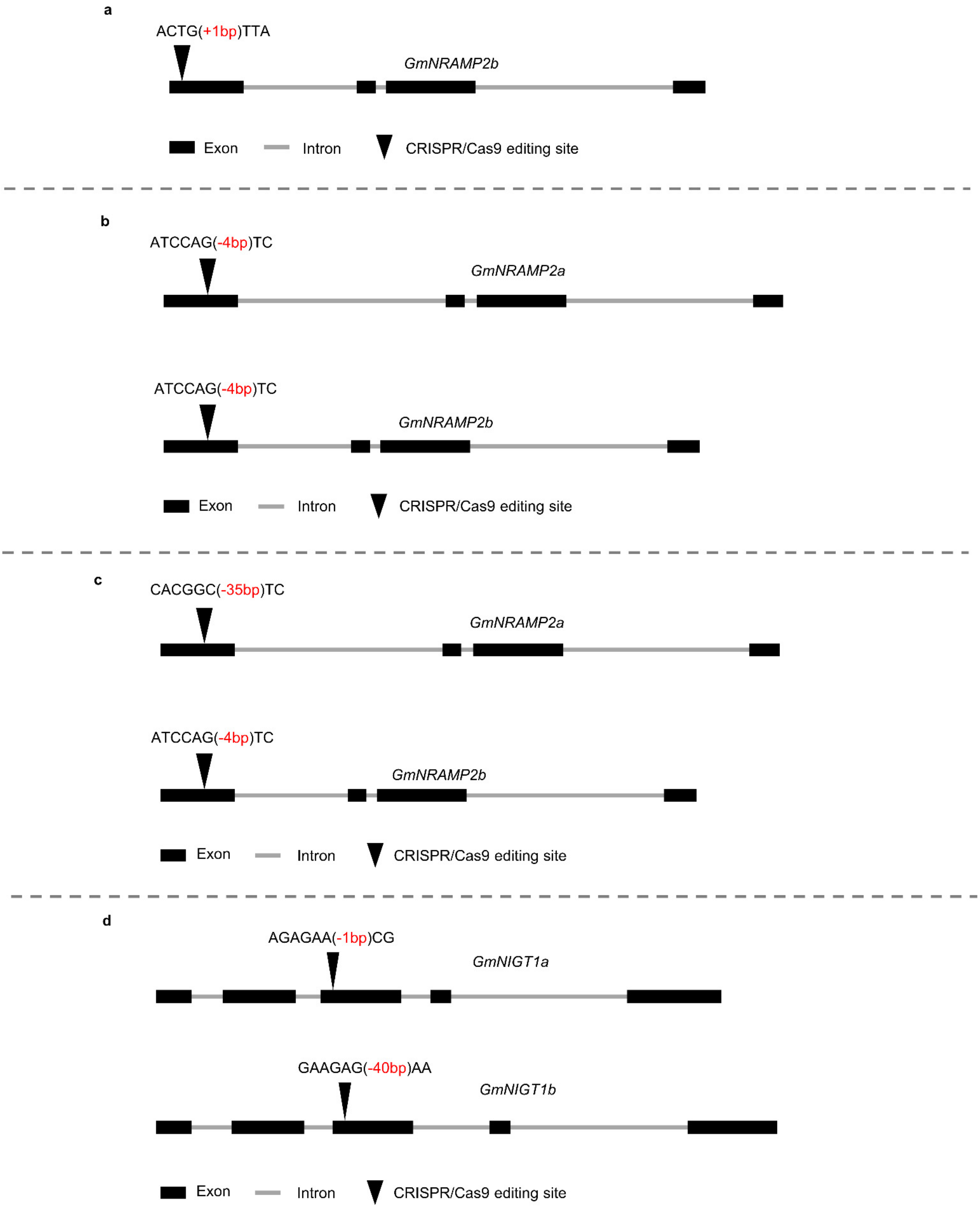

Supplementary Fig. 7. Mutation sites of various genetic materials. (**a**) *nramp2b* single mutant. (**b-c**) Two independent *nramp2ab* double mutants. (**d**) *nigt1ab* double mutant. CRIPSR/Cas9 editing sites are indicated with triangles.

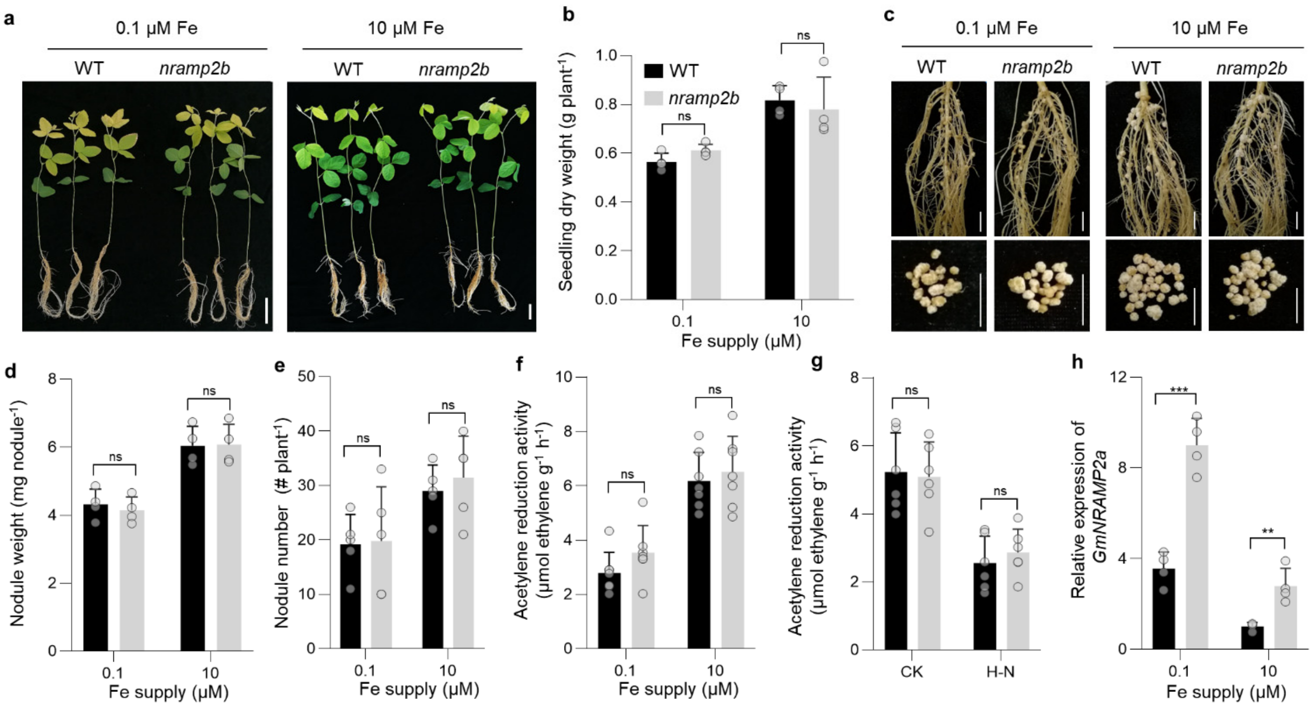

Supplementary Fig. 8. Phenotypic analysis of *nramp2b* mutant.

(**a-e**) Comparison of seedling and nodule growth in WT and *nramp2b* mutant under symbiotic conditions. (**f-g**) Acetylene reduction activity. (**h**) Expression of *GmNRAMP2a* in *nramp2b* mutant. Relative expression levels were determined by real-time RT-PCR. *EF-1α* was used as an internal standard. Seedlings at 11 dpi were transplanted to a low-N nutrient solution with 0.1 or 10 μM $FeSO_4$ for 9 d (**a-f, h**). Nodules at 20 dpi were treated with low-N (CK) or high-N (H-N) for 2 d (**g**). Data are means + SD. n = 4 (**b**, **d**), 5 (**e**), 7 (**f**), 6 (**g**), 4 (**h**) biologically independent replicates. Asterisks show significant differences compared with WT: $0.001<^{**}P\leq 0.01$, $^{***}P\leq 0.001$ by Student's t-test, two-tailed *t*-test. Scale bars = 10 (**a**), 1 (**c**) cm.

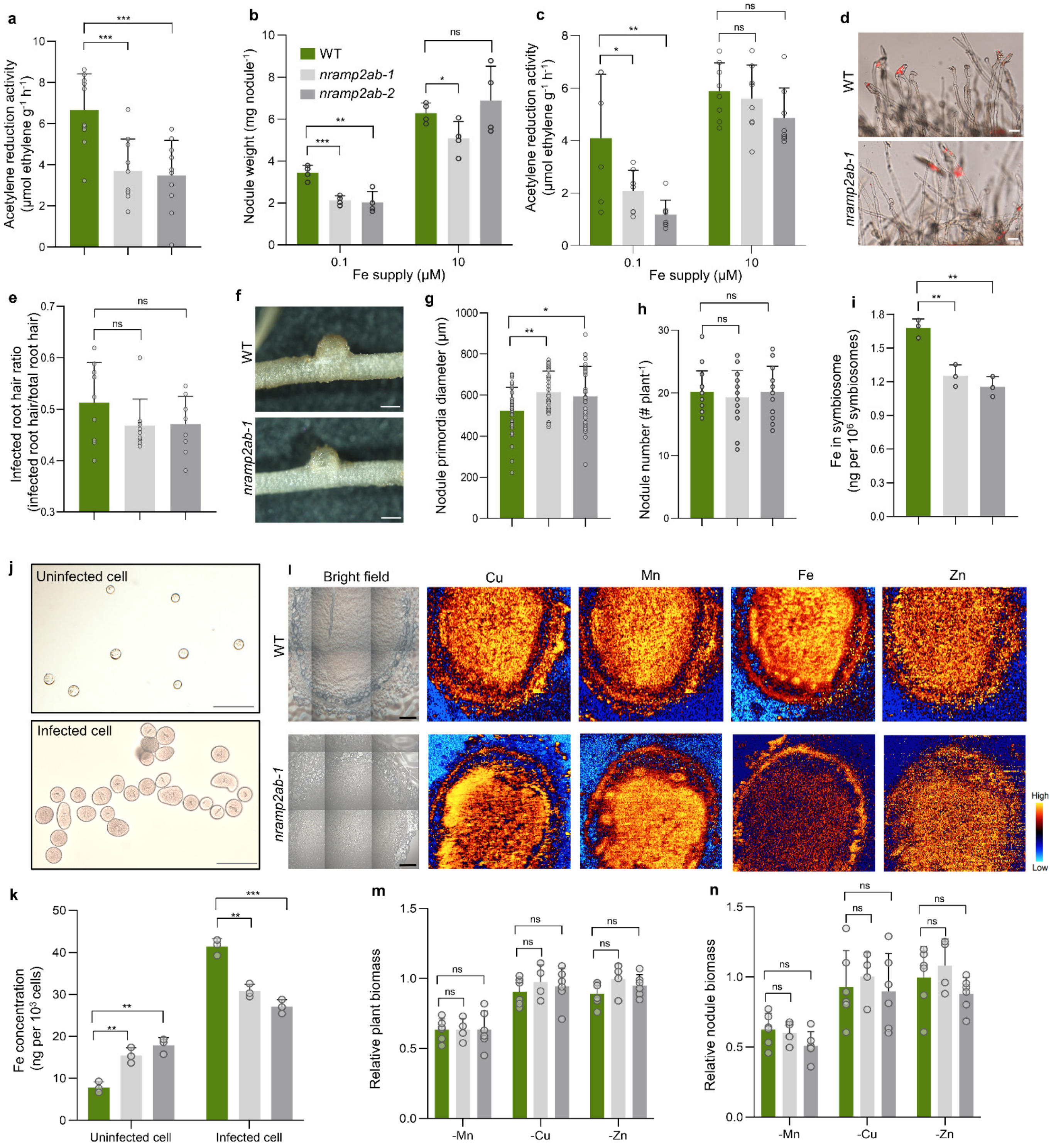

Supplementary Fig. 9. Phenotypic analysis of *nramp2ab* mutants.

(**a**) Acetylene reduction activity. 4-d-old seedlings were inoculated and cultivated in low-N solution for 22 d. (**b**-**c**) Comparison of nodule weight (**b**) and acetylene reduction activity (**c**) in WT and mutants under Fe-deficient or -sufficient conditions. Seedlings at 11 dpi were transplanted to a low-N solution with 0.1 or 10 μM $FeSO_4$ for 9 d.

(**d**-**e**) Rhizobia infection in root hair. The ratio of infected root hairs was calculated and shown in (**e**). Scale bars,100 μm. (**f**-**g**) nodule primordium in roots. The diameter of nodule primordium was calculated and shown in (**g**). Scale bars, 500 μm. Soybean seedlings were inoculated with an RFP-labeled rhizobia strain. Seedlings at 4 dpi were used for root hair observation, and seedlings at 7 dpi were used for nodule primordium observation.

(**h**) Nodule number per plant. (**i**) Fe content in the symbiosomes of nodules. Nodules at 22 dpi were sampled, and intact symbiosomes were isolated by percoll gradient centrifugation.

(**j**-**k**) Isolation (**j**) and Fe determination (**k**) of infected and uninfected cells from nodules. Scale bars, 100 μm. Nodules at 22 dpi were sampled, and intact infected and uninfected cells were isolated by digestion with cellulase and macerozyme. ICP-MS was used

for Fe determination.

(**l**) LA-ICP-TOF-MS elemental mapping of nodule cross-section. Scale bars, 200 μm. Nodules at 22 dpi were sampled for analysis.

(**m**-**n)** Relative biomass of seedling and nodule after trace element deficiencies. Seedlings at 7 dpi were transplanted to a low-N nutrient solution with Mn, Cu or Zn deficiency for 18 d, and the biomass per plant and per nodule were weighed. The value relative to metal-sufficient conditions is shown. Data are means + SD. n = 10 (**a**), 4 (**b**), 6-8 (**c**), 9 (**e**), 33 (**g**), 16 (**h**), 4-6 (**m**-**n**) biologically independent replicates, or 3 (**i**, **k**) independent pools (1 g of nodules per pool). Asterisks show significant differences compared with WT: $0.01<*P\leq 0.05$, $0.001<**P\leq 0.01$, $***P\leq 0.001$ by Student's *t*-test, two-tailed *t*-test.

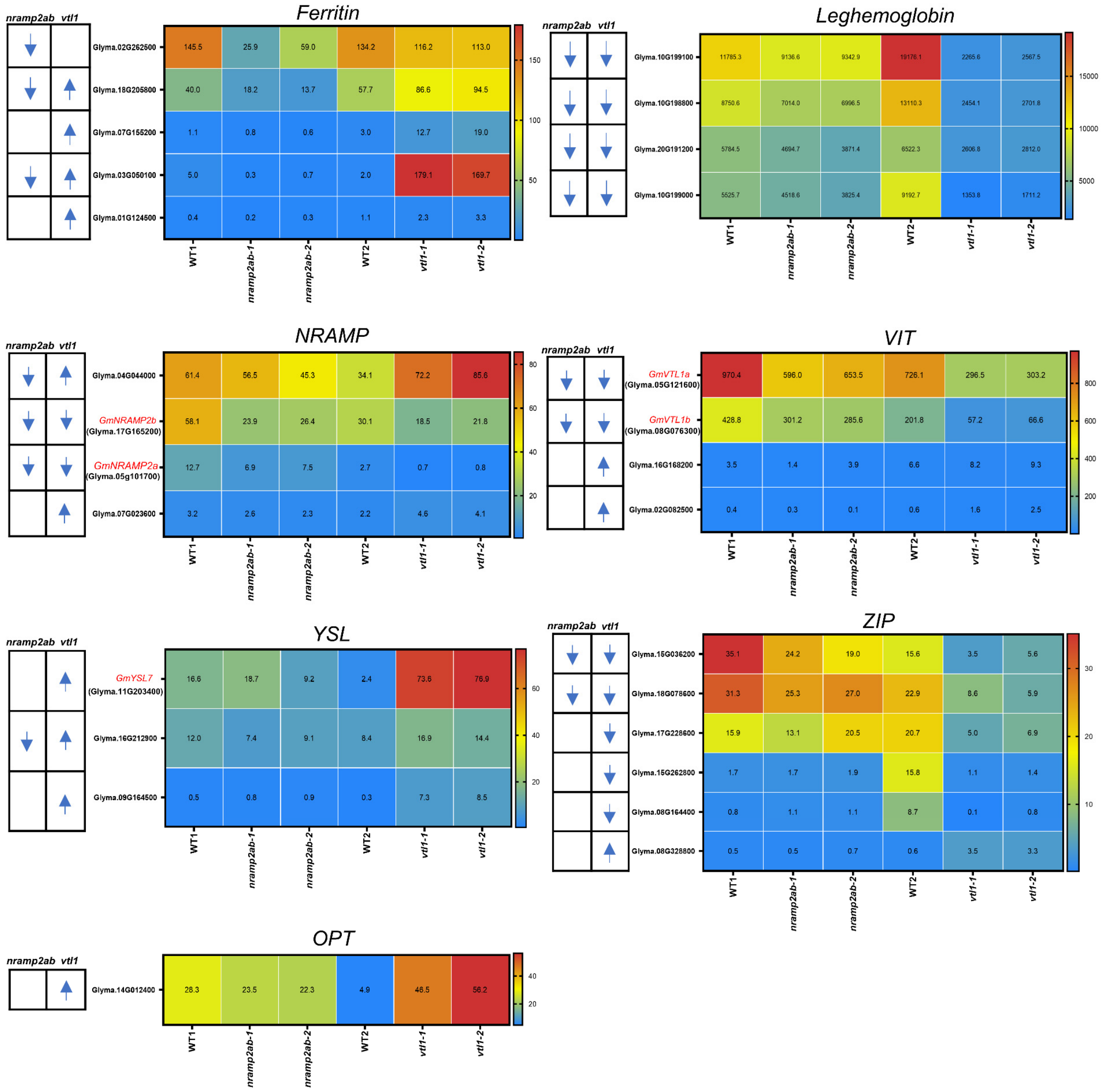

Supplementary Fig. 10. Expression profiles of Fe-related gene families in *nramp2ab* and *vtl1* mutants. Nodules collected at 21 dpi from WT, *nramp2ab*, and *vtl1* mutants were used for RNA-seq analysis. FPKM (Fragments Per Kilobase of transcript per Million mapped reads) values are shown. The arrows in the left box of the heatmap indicate whether the gene is upregulated (upward) or downregulated (downward) in the mutant (*nramp2ab* or *vtl1*), in comparison to the WT. NRAMP, natural resistance-associated macrophage protein; VIT, vacuolar iron transporter; YSL, yellow stripe-like protein; ZIP, Zrt-, Irt-like protein; OPT, oligopeptide transporter. WT1, the wild-type control for *nramp2ab* mutants. WT2, the wild-type control for *vtl1* mutants.

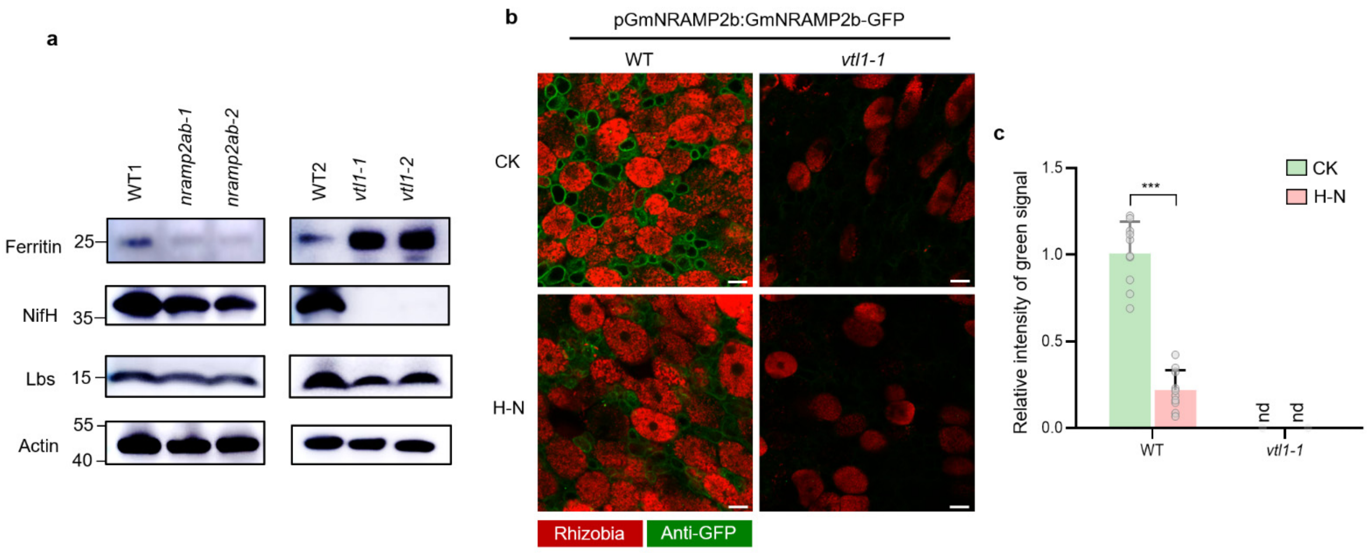

Supplementary Fig. 11. Expression levels of Fe-related proteins in *nramp2ab* and *vtl1* mutants.

(**a**) Western blot of Ferritin, NifH, leghemoglobins (Lbs) and actin in nodules. Nodules collected at 21 dpi from WT, *nramp2ab*, and *vtl1* mutants were used for analysis. WT1, the wild-type control for *nramp2ab* mutants. WT2, the wild-type control for *vtl1* mutants. Actin was used for internal control. (**b-c**) Immunostaining staining of transgenic nodules expressing *pGmNRAMP2b:GmNRAMP2b-GFP* in *vtl1-1* mutant. Transgenic nodules carrying *pGmNRAMP2b:GmNRAMP2b-GFP* from WT and *vtl1-1* hairy roots at 17 dpi were treated with high-N (H-N) for 1 d. Five independent transgenic lines were investigated and consistent results were obtained, with one representative image presented in (**b**). nd means non-detected values. Scale bars, 20 µm. Data are means + SD. n = 10 (**c**) replicates from independent nodules. Asterisks in (**c**) indicate significant differences compared with CK: ***$P \leq 0.001$ by Student's *t*-test, two-tailed *t*-test.

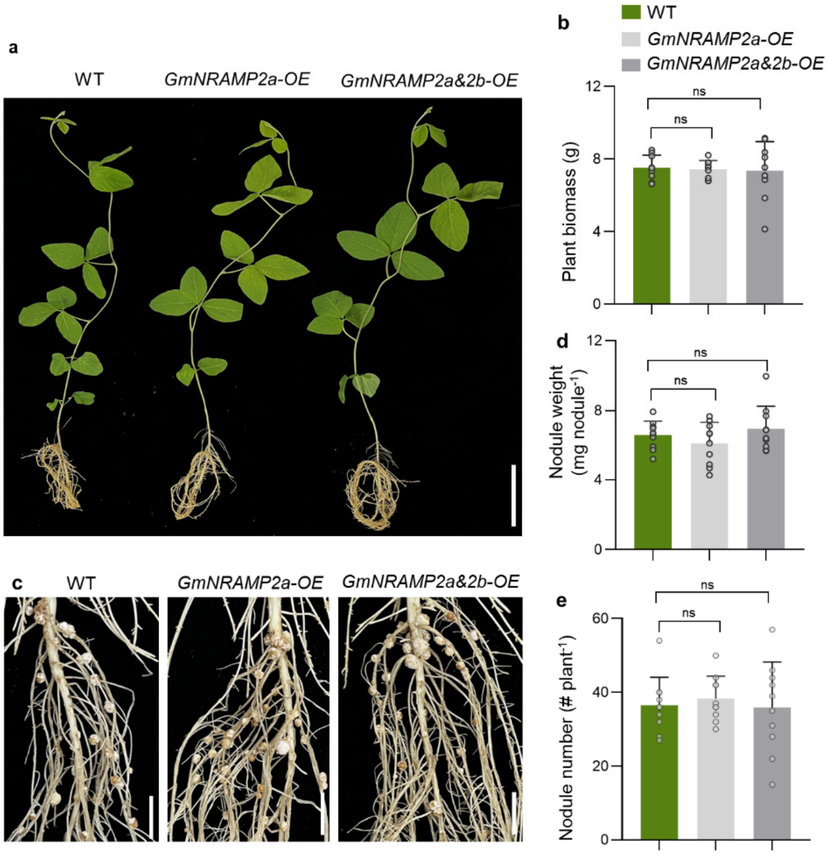

Supplementary Fig. 12. Comparison of seedling and nodule growth in *GmNRAMP2a&2b* overexpression lines and their segregated WT.

(**a**) seedling growth state. (**b**) plant biomass. (**c**) nodule growth state. (**d**) single nodule weight. (**e**) nodule number per plant. 4-d-old seedlings were inoculated with rhizobia and grown in a low-N nutrient solution for 21 d. Data are means + SD. n = 9 (**b**), 10 (**d**, **e**) biologically independent replicates. Scale bars = 10 (**a**), 1 (**c**) cm.

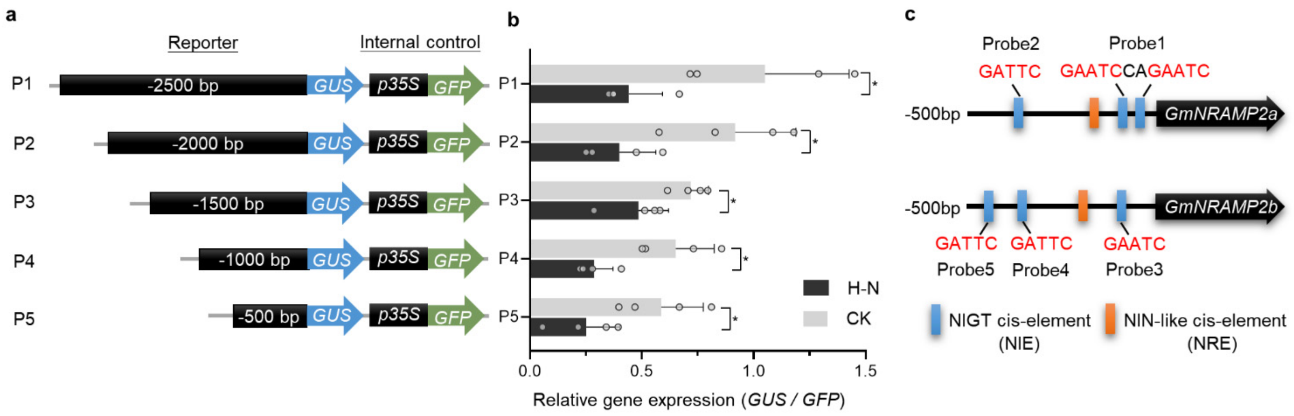

d

Gene	*Cis*-acting element	Abbreviation	Trans-acting factor	Promoter position
GmNRAMP2a	GAATC / GATTC	NIE	NIGT	-32, -39, -353
	CACTTCAGTCCTCTGAAGAT	NRE$_{GmNRAMP2a}$	NIN/NLP	-94
GmNRAMP2b	GAATC / GATTC	NIE	NIGT	-47, -374, -442
	CCTTTCAAGTCACAAGCT	NRE$_{GmNRAMP2b}$	NIN/NLP	-158
AtNIR1	CCCTTTACATTGCTCAAGAG	NRE$_{AtNIR1}$	NIN/NLP	-91
AtNRT2.4	GAATC / GATTC	NIE	NIGT	-116, -138, -192
	GAATATTC		NIGT	-261

Supplementary Fig. 13. Discovery of N-responsive *cis*-acting elements in the promoter of *GmNRAMP2a&2b*.

(**a**-**b**) Promoter segmentation expression assay. Five segments of *GmNRAMP2b* promoter were fused with the reporter gene *GUS* individually (**a**), and introduced into soybean hair roots for *GUS* expression assay (**b**). Transgenic nodules at 17 dpi from hairy roots were treated with or without (CK, control group) high-N (H-N) solution for 1 d, and then were collected for RNA extraction. Expression levels relative to *GFP* (internal control) expression are shown. Data are means + SD. n = 4 biologically independent replicates. (**c**) Predicted NIGT and NIN-like *cis*-acting elements (NIE and NRE) in the promoters of *GmNRAMP2a&2b*. (**d**) Sequence of *cis*-acting elements for NIGT and NIN/NLP. Red sequences indicate the predicted conserved nucleotides.

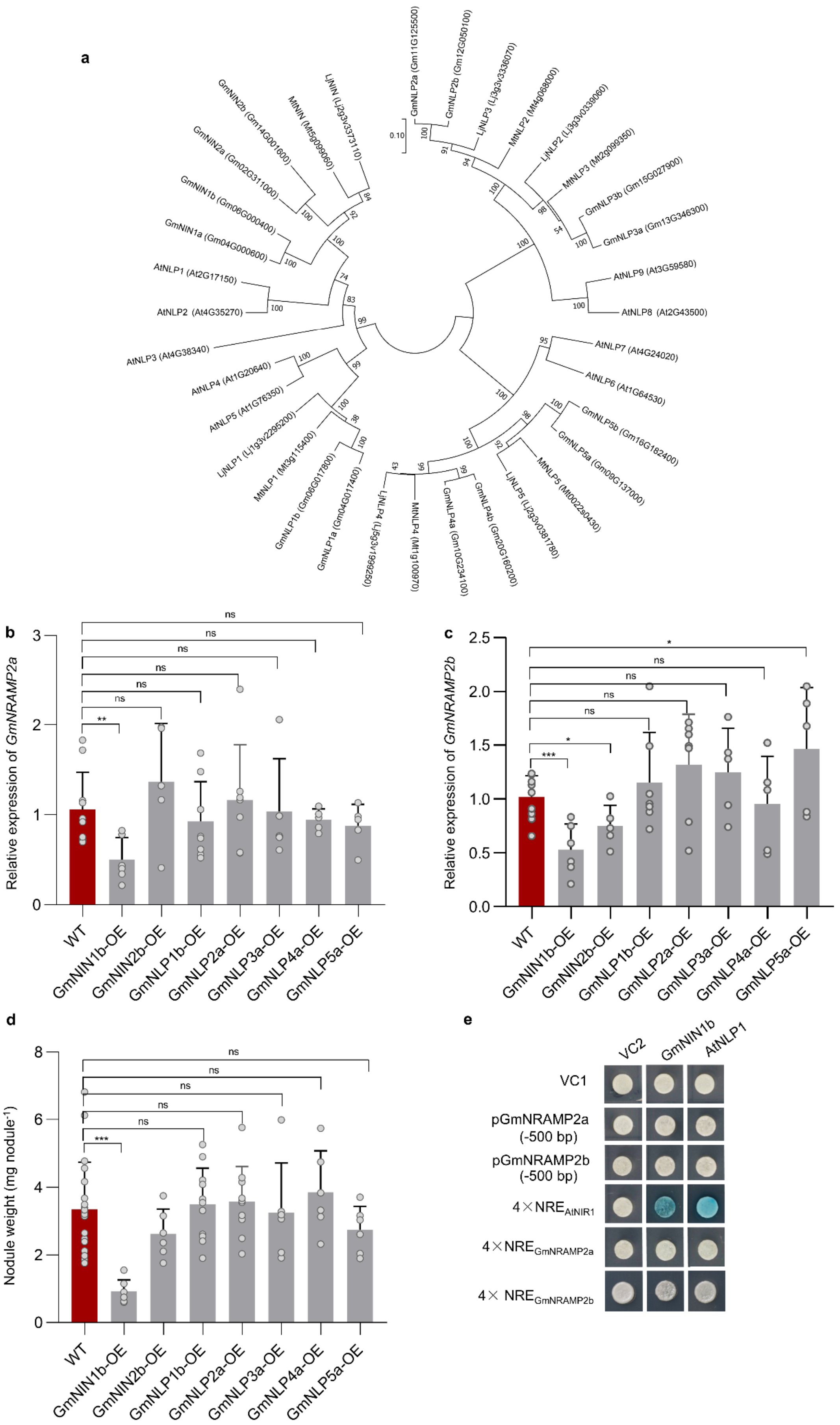

Supplementary Fig. 14. Effects of GmNINs/GmNLPs on the expression of *GmNRAMP2a&2b* in soybean.

(**a**) Phylogenetic tree of NIN/NLP family members in plants. NIN/NLP protein sequences of Arabidopsis (At), soybean (Gm), and Medicago (Mt) were obtained from Phytozome (phytozome.jgi.doe.gov), and NIN/NLP protein sequences of *Lotus japonicus* (Lj) were obtained from miyakogusa.jp (kazusa.or.jp/lotus). The phylogenetic tree was constructed with MEGA7 using the Neighbor-

Joining method and 2000 bootstrap replicates. The evolutionary distances were computed using the Poisson correction method and are in the units of the number of amino acid substitutions per site.

(**b**-**c**) Altered expression of *GmNRAMP2a* (**b**) and *GmNRAMP2b* (**c**) by *GmNINs/GmNLPs* overexpression in nodules. Relative expression levels were determined by real-time RT-PCR. *EF-1α* was used as an internal standard. n = 5-10 biologically independent replicates. (**d**) Single nodule weight. n = 6-20 biologically independent replicates. Transgenic nodules overexpressing *GmNINs/GmNLPs* at 17 dpi from hairy roots were sampled for RNA extraction and nodule weight determination. Data are means + SD. Asterisks show significant differences compared with WT: $0.001<^{**}P\leq 0.01$, $^{***}P\leq 0.001$ by Student's *t*-test, two-tailed *t*-test. (**e**) Yeast one-hybrid assay. The effectors (GmNIN1b, AtNLP1) combined with the reporters (pGmNRAMP2a, pGmNRAMP2b, $4\times NRE_{AtNIR1}$, $4\times NRE_{GmNRAMP2a}$, $4\times NRE_{GmNRAMP2b}$) were introduced into yeast strain EGY48 and cultured on SD medium (-Trp-Ura) containing X-gal at 30°C. VC1, pB42AD. VC2, pLacZi.

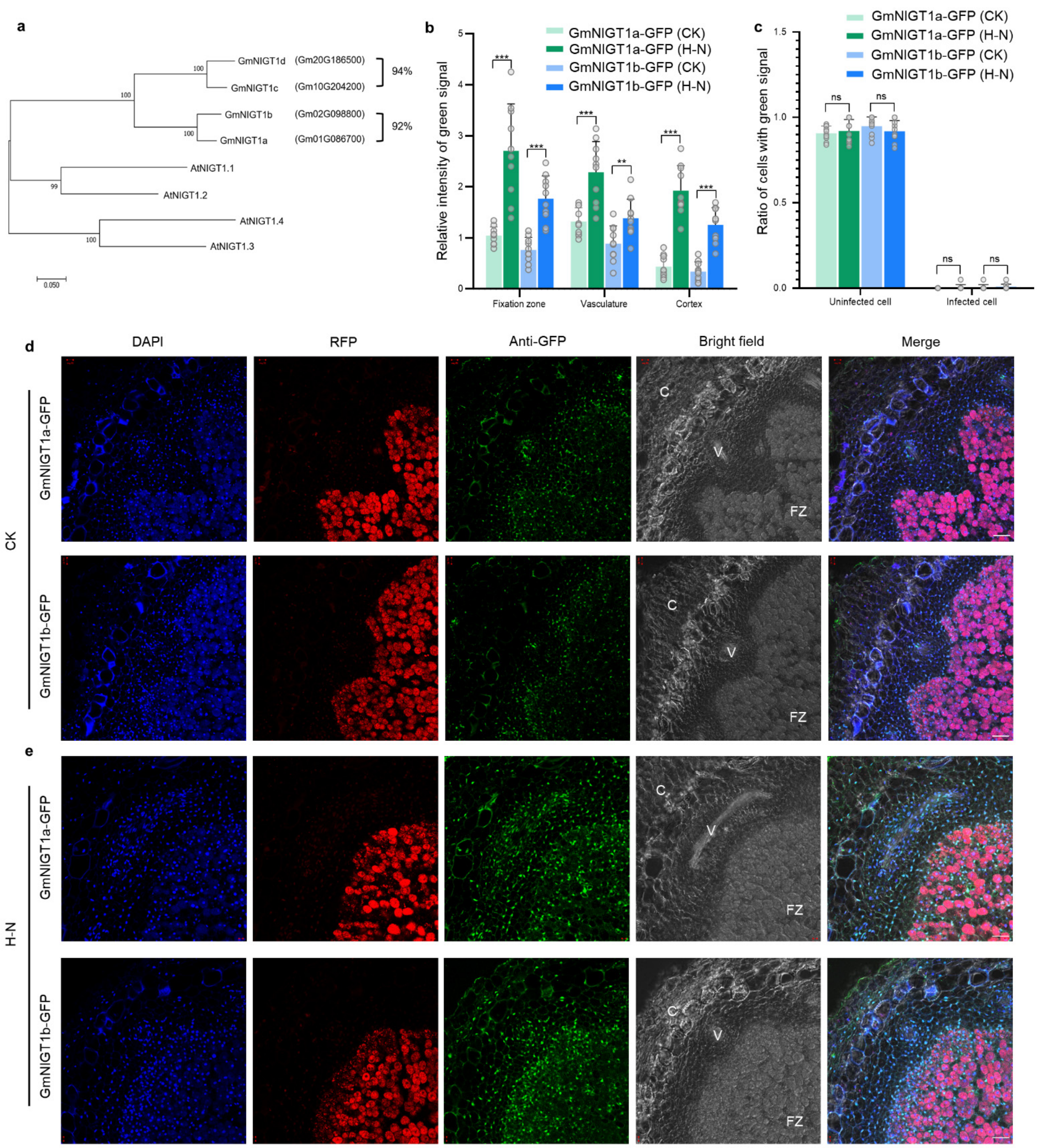

Supplementary Fig. 15. Protein abundance of GmNIGT1a&1b in response to N availability.

(**a**) Phylogenetic tree of NIGT family members in soybean and Arabidopsis. Protein sequences were obtained from Phytozome. The phylogenetic tree was constructed with MEGA7 using the Neighbor-Joining method and 2000 bootstrap replicates. The evolutionary distances were computed using the Poisson correction method and are in the units of the number of amino acid substitutions per site. Gm, soybean; At, Arabidopsis. The percentage value indicates the protein similarity between them.

(**b**-**e**) Immunostaining of *pGmNIGT1a/1b: GmNIGT1a/1b-GFP* transgenic nodules under CK (**d**) or H-N (**e**) conditions. **b**, Relative intensity of green signal from (**d**, **e**). **c**, Ratio of cells with green signal in (**d**, **e**). Transgenic nodules at 17 dpi from hairy roots were treated with (H-N) or without (CK) high-N solution for 1 d, and then used for immunostaining. Blue shows signals from nucleus; Red shows RFP-tagged rhizobia; Green shows anti-GFP signals. C, cortex; V, vasculature; FZ, fixation zone. Scale bars, 50 µm. Data are means + SD. n = 10 (**b**, **c**) replicates from 10 independent nodules. Asterisks in (**b**) indicate significant differences compared with their respective CK: 0.001<**$P \leq 0.01$, ***$P \leq 0.001$ by Student's *t*-test, two-tailed *t*-test.

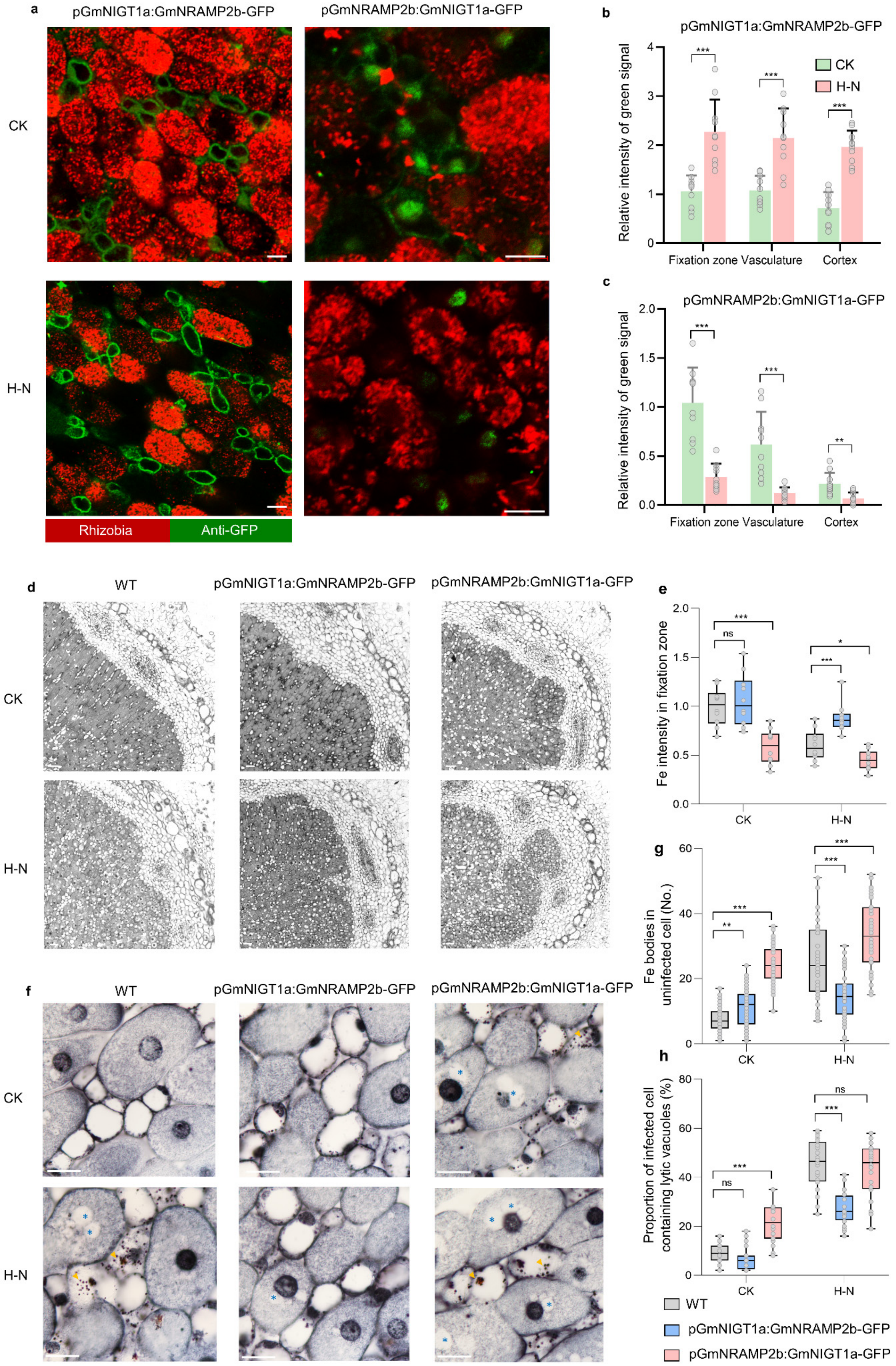

Supplementary Fig. 16. The impact of *in situ* overexpression of *GmNRAMP2b* and *GmNIGT1a* on Fe homeostasis in nodules. (**a-c**) Protein abundance response to high-N. Red shows RFP-tagged rhizobia; Green shows anti-GFP signals. Transgenic nodules from hairy roots carrying *pGmNIGT1a:GmNRAMP2b-GFP* (**b**) or *pGmNRAMP2b:GmNIGT1a-GFP* (**c**) at 17 dpi were transplanted to high-N (H-N) for 2 d. Five independent transgenic lines were investigated and consistent results were obtained, with one

representative image presented in (**a**). Scale bars, 10 μm.

(**d**-**h**) Fe accumulation and distribution in nodules, including (**d-e**) Nodule Fe intensity, (**f-g**) Fe bodies in uninfected cell, and (**h**) lytic vacuoles in infected cell. Nodule samples were sectioned and stained with Perls solution, followed by DAB intensification. Yellow arrows and blue asterisks in (**f**) indicate Fe bodies and lytic vacuoles respectively. Scale bars = 1 mm (**d**), 50 μm (**f**).

The boxes in (**b, c, e, g, h**) indicate the first and third quartiles, and the whiskers indicate the minimum and maximum values. The lines within the boxes indicate the median values. n = 10 (**b**, **c**, **e**), 50 (**g**), or 20 (**h**) biologically independent replicates. Asterisks show significant differences compared with WT: $0.01<{}^{*}P\leq 0.05$, $0.001<{}^{**}P\leq 0.01$, $^{***}P\leq 0.001$ by Student's *t*-test, two-tailed *t*-test.

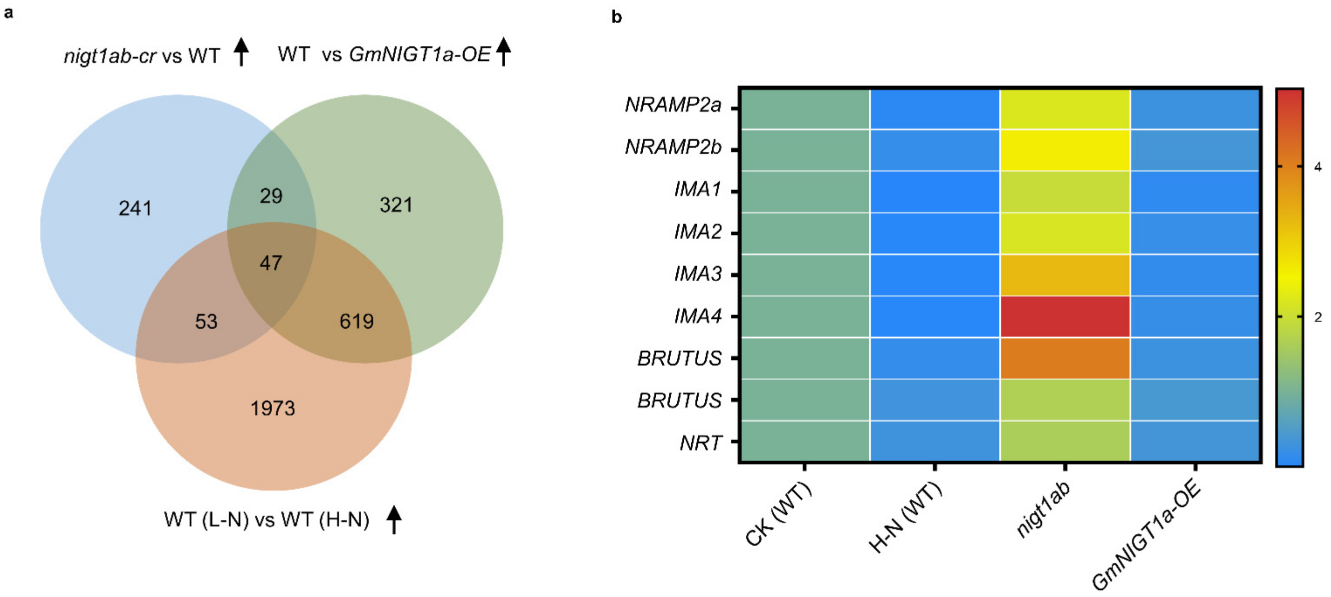

Supplementary Fig. 17. Putative downstream genes regulated by GmNIGT1a&1b.

(**a**) Venn diagram showing the genes potentially regulated by GmNIGT1a&1b. Nodules grown in low-N (L-N) solution at 21 dpi from wild type (WT), knockout (*nigt1ab-cr*) and overexpression (*GmNIGT1a-OE*) lines were used for RNA-seq analysis. Nodules at 20 dpi treated with high-N (H-N) for 1 d were used for RNA-seq analysis. Upward arrows indicate upregulated genes with fold change larger than 1.5.

(**b**) Heatmap showing the relative transcriptional levels of *NRAMP2s*, *IMAs*, *BURTUSs* and *NRT*. The values for each gene represent the fold change in FPKM values of nodules compared to their respective control (CK) in the wild type (WT).
