# Supplementary material for: Inorganic nitrogen inhibits symbiotic nitrogen fixation through blocking NRAMP2-mediated iron delivery in soybean nodules
Source: Nat Commun. 2024 Oct 17;15:8946. doi: 10.1038/s41467-024-53325-y (PMC11484902; doi:10.1038/s41467-024-53325-y)
Supplement: Supplementary file 3 — Description of additional supplementary files [file 41467_2024_53325_MOESM3_ESM.pdf]

Description of Additional Supplementary Files

Supplementary Data 1: Differently expressed genes by -Fe and H-N

Supplementary Data 2: Comparative RNA-seq data of WT, vtl1 and nramp2 mutants

Supplementary Data 3: Comparative RNA-seq data of WT, nigt1ab and NIGT1a-OE line

Supplementary Data 4: Primers used in this study
