# Supplementary material for: Inorganic nitrogen inhibits symbiotic nitrogen fixation through blocking NRAMP2-mediated iron delivery in soybean nodules
Source: Nat Commun. 2024 Oct 17;15:8946. doi: 10.1038/s41467-024-53325-y (PMC11484902; doi:10.1038/s41467-024-53325-y)
Supplement: Supplementary file 8 — Reporting Summary [file 41467_2024_53325_MOESM8_ESM.pdf]

nature portfolio

Corresponding author(s): Zhi-Chang Chen, De-Qiang Duanmu, Yue-Feng Guan

Last updated by author(s): Sep 21, 2024

Reporting Summary

Nature Portfolio wishes to improve the reproducibility of the work that we publish. This form provides structure for consistency and transparency in reporting. For further information on Nature Portfolio policies, see our Editorial Policies and the Editorial Policy Checklist.

Statistics

For all statistical analyses, confirm that the following items are present in the figure legend, table legend, main text, or Methods section.

n/a	Confirmed	
☐	☒	The exact sample size (*n*) for each experimental group/condition, given as a discrete number and unit of measurement
☐	☒	A statement on whether measurements were taken from distinct samples or whether the same sample was measured repeatedly
☐	☒	The statistical test(s) used AND whether they are one- or two-sided<br>*Only common tests should be described solely by name; describe more complex techniques in the Methods section.*
☒	☐	A description of all covariates tested
☒	☐	A description of any assumptions or corrections, such as tests of normality and adjustment for multiple comparisons
☐	☒	A full description of the statistical parameters including central tendency (e.g. means) or other basic estimates (e.g. regression coefficient) AND variation (e.g. standard deviation) or associated estimates of uncertainty (e.g. confidence intervals)
☐	☒	For null hypothesis testing, the test statistic (e.g. *F*, *t*, *r*) with confidence intervals, effect sizes, degrees of freedom and *P* value noted<br>*Give P values as exact values whenever suitable.*
☒	☐	For Bayesian analysis, information on the choice of priors and Markov chain Monte Carlo settings
☒	☐	For hierarchical and complex designs, identification of the appropriate level for tests and full reporting of outcomes
☒	☐	Estimates of effect sizes (e.g. Cohen's *d*, Pearson's *r*), indicating how they were calculated

Our web collection on statistics for biologists contains articles on many of the points above.

Software and code

Policy information about availability of computer code

Data collection	ZEN2 was used to collect fluorescence signals. LightCycler 96 Software (Version 1.1.0.1320, Roche, Germany) was used to collect real-time PCR data. MassHunter 4.3 Workstation Software was used to collect ICP-MS data. Phytozome database (https://phytozome.jgi.doe.gov/) was used to retrieve the gene's expression levels and annotations.
Data analysis	Microsoft Excel 2013 and GraphPad Prism 8 were used to analyze the data; PowerPoint 2016 was used for Figure assembly. Image J (v1.8.0) was used to analyze protein abundance; IBM SPSS Statistics 19 was used for statistical analysis.

For manuscripts utilizing custom algorithms or software that are central to the research but not yet described in published literature, software must be made available to editors and reviewers. We strongly encourage code deposition in a community repository (e.g. GitHub). See the Nature Portfolio guidelines for submitting code & software for further information.

Data

Policy information about availability of data

All manuscripts must include a data availability statement. This statement should provide the following information, where applicable:

- Accession codes, unique identifiers, or web links for publicly available datasets
- A description of any restrictions on data availability
- For clinical datasets or third party data, please ensure that the statement adheres to our policy

All data are available in the manuscript or the Supplementary Materials. RNA-seq data have been deposited at NCBI (National Center for Biotechnology Information, project accession number PRJNA875247).

Research involving human participants, their data, or biological material

Policy information about studies with human participants or human data. See also policy information about sex, gender (identity/presentation), and sexual orientation and race, ethnicity and racism.

Reporting on sex and gender	*Use the terms sex (biological attribute) and gender (shaped by social and cultural circumstances) carefully in order to avoid confusing both terms. Indicate if findings apply to only one sex or gender; describe whether sex and gender were considered in study design; whether sex and/or gender was determined based on self-reporting or assigned and methods used. Provide in the source data disaggregated sex and gender data, where this information has been collected, and if consent has been obtained for sharing of individual-level data; provide overall numbers in this Reporting Summary. Please state if this information has not been collected. Report sex- and gender-based analyses where performed, justify reasons for lack of sex- and gender-based analysis.*
Reporting on race, ethnicity, or other socially relevant groupings	*Please specify the socially constructed or socially relevant categorization variable(s) used in your manuscript and explain why they were used. Please note that such variables should not be used as proxies for other socially constructed/relevant variables (for example, race or ethnicity should not be used as a proxy for socioeconomic status). Provide clear definitions of the relevant terms used, how they were provided (by the participants/respondents, the researchers, or third parties), and the method(s) used to classify people into the different categories (e.g. self-report, census or administrative data, social media data, etc.) Please provide details about how you controlled for confounding variables in your analyses.*
Population characteristics	*Describe the covariate-relevant population characteristics of the human research participants (e.g. age, genotypic information, past and current diagnosis and treatment categories). If you filled out the behavioural & social sciences study design questions and have nothing to add here, write "See above."*
Recruitment	*Describe how participants were recruited. Outline any potential self-selection bias or other biases that may be present and how these are likely to impact results.*
Ethics oversight	*Identify the organization(s) that approved the study protocol.*

Note that full information on the approval of the study protocol must also be provided in the manuscript.

Field-specific reporting

Please select the one below that is the best fit for your research. If you are not sure, read the appropriate sections before making your selection.

☒ Life sciences ☐ Behavioural & social sciences ☐ Ecological, evolutionary & environmental sciences

For a reference copy of the document with all sections, see nature.com/documents/nr-reporting-summary-flat.pdf

Life sciences study design

All studies must disclose on these points even when the disclosure is negative.

Sample size	Sample sizes were chosen based on our experience on the experimental variability of this type of experiment and the desire to get statistically significant data to support meaningful conclusions. At least three biologically independent samples were used to derive statistics. The number of independent biological seedlings or replicates has been shown in each figure legend.
Data exclusions	No data were excluded
Replication	Each experiment was repeated at least two times, and similar results were obtained.
Randomization	Seedlings were grown randomly in the growth chamber.
Blinding	Experiments were not blinded. Data were always collected according to the genotype of plants.

Reporting for specific materials, systems and methods

We require information from authors about some types of materials, experimental systems and methods used in many studies. Here, indicate whether each material, system or method listed is relevant to your study. If you are not sure if a list item applies to your research, read the appropriate section before selecting a response.

Materials & experimental systems

n/a	Involved in the study
☐	☒ Antibodies
☐	☒ Eukaryotic cell lines
☒	☐ Palaeontology and archaeology
☒	☐ Animals and other organisms
☒	☐ Clinical data
☒	☐ Dual use research of concern
☐	☒ Plants

Methods

n/a	Involved in the study
☒	☐ ChIP-seq
☒	☐ Flow cytometry
☒	☐ MRI-based neuroimaging

Antibodies

Antibodies used

Primary antibody
Anti-green fluorescent protein; Thermo Fisher Scientific; Cat. No. A11122; dilution 1: 1,000
Anti-Actin (plant specific); ABclonal; Cat. No. AC009; dilution 1:5,000
Anti-Ferritin 1-2 (plant); AgriSera; Cat. No. AS15 2898; dilution 1:2,500
Anti-Nitrogenase (NifH); AgriSera; Cat. No. AS01 021A; dilution 1:2,000
Anti-green fluorescent protein; TransGen; HT801; dilution 1: 1,000
Anti- ALP; Abcam; Cat. No. ab113688; dilution 1:1,000
Anti-PGK1; Abcam; Cat. No. ab113687; dilution 1:2,000
Anti-porin; Abcam; Cat. No. ab110326; dilution 1:1,000
V-ATPase; AgriSera; Cat. No. AS07 213; dilution 1:2,000
H+-ATPase; AgriSera; Cat. No. AS07 260; dilution 1:2,000

Secondary antibody
Alexa Fluor™ 488 Goat anti-Rabbit IgG; Thermo Fisher Scientific; Cat. No. A-11008; dilution 1:2,000
Alexa Fluor™ 555 Goat anti-Rabbit IgG; Thermo Fisher Scientific; Cat. No. A-21428; dilution 1:2,000
ProteinFond® Goat Anti-Mouse IgG; TransGen; Cat. No. HS201-01; dilution 1:5,000
Goat Anti-Rabbit IgG; Biosharp; Cat. No. BL003A; dilution 1:5,000
Rabbit Anti-Goat IgG/HRP; Solarib; Cat. No. SE238; dilution 1:1,000
Goat anti-Chicken IgY; Thermo Fisher Scientific; Cat. No. A16054; dilution 1:10,000

Validation

Primary antibody: Anti-green fluorescent protein; Thermo Fisher Scientific; Cat. No. A11122; dilution 1:1,000
Anti-GFP was validated for a variety of applications such as western analysis, immunocytochemistry, immunoprecipitation, flow cytometry and immunohistochemistry. Anti-GFP was validated in Chimpanzee, Dog, Fish, Fruit fly, Hamster, Human, Mouse, Non-human primate, Pig, Rat, Sheep, Tag, Zebrafish. More details can be found at online website: (https://www.thermofisher.com.cn/zh/antibody/product/GFP-Antibody-Polyclonal/A-11122).

Primary antibody: Anti-Actin (plant specific); Abclonal; Cat. No. A009; dilution 1:15,000
Anti-Actin was validated for a variety of applications such as western blot, Co-Immunoprecipitation, Chromatin Immunoprecipitation and immunoblotting. Anti-Actin was validated in Zea mays, Arabidopsis thaliana, Tomato, Brassica napus, Nicotiana tabacum, Lycopersicon esculentum, Glycine max, Triticium aestivum, Mus musculus, Solanum tuberosum, Nicotiana benthamiana, Paeonia suffruticosa. More details can be found at online website: (https://ap.abclonal.com/catalog-antibodies/ActinplantspecificMouseAb/AC009).

Primary antibody: Anti-Ferritin 1-2 (plant); Agrisera; Cat. No. AS15 2898; dilution 1:2,500
Anti-Ferritin has been validated for Western blot. Anti-Ferritin was validated in A. thaliana, B. oleracea, H. vulgare, M. truncatula, S. oleracea, P. sativum. More details can be found at online website: (https://www.agrisera.com/en/artiklar/ferritin-plant-2.html).

Primary antibody: Anti-Nitrogenase (NifH); Agrisera; Cat. No. AS01 021A; dilution 1:2,000
Anti-NifH was validated for a variety of applications such as Immunofluorescence, Immunohistochemistry, western blot. Anti-NifH was validated in Anabaena PCC7120, Clostridium butyricum, Codakikras, Cylindrospemopsis raciborskii CS-505, Dolichospermum sp., Nostoc sp, Rhodopseudomonas palustris, Trichodesmium sp., nodules of Trifolium repens L., Vibrio natriegens ATCC 14048. More details can be found at online website: (https://www.agrisera.com/en/artiklar/nitrogenase-nif-global-antibody.html).

Primary antibody: Anti-green fluorescent protein; TransGen; HT801; dilution 1:1,000
Anti-GFP has been validated for Western blot. More details can be found at online website: (https://www.transgen.com/antibody_tag/390.html)

Primary antibody: Anti- ALP; Abcam; Cat. No. ab113688; dilution 1:1,000
Anti-ALP was suitable for WB, ICC, IF and reacts with Saccharomyces cerevisiae. More details can be found at online website: (https://www.abcam.com/products/primary-antibodies/jho8-antibody-1d3a10-ab113688.html).

Primary antibody: Anti-PGK1; Abcam; Cat. No. ab113687; dilution 1:2,000
Anti-PGK1 was suitable for WB and reacts with Saccharomyces cerevisiae. More details can be found at online website: (https://www.abcam.com/products/primary-antibodies/pgk1-antibody-22c5d8-ab113687.html?productWallTab=ShowAll).

Primary antibody: Anti-porin; Abcam; Cat. No. ab110326; dilution 1:1,000
Anti-porin was suitable for WB and reacts with Saccharomyces cerevisiae. More details can be found at online website: (https://www.abcam.com/products/primary-antibodies/vdac1porin-antibody-16g9e6bc4-mitochondrial-loading-control-ab110326.html).

Primary antibody: V-ATPase; Agrisera; Cat. No. AS07 213; dilution 1:2,000
V-ATPase was suitable for WB, IF, IHC and reacts with higher plants including A.comosus, A.thaliana, C.sativus, C. australis R.Br, C.reinhardtii, F. margarita Swingle, H.vulgare, L.esculentum, L.longiflorum, Malus × domestica Borkh. c.v. Fuji, M. truncatula, M.crystalliunum, N.tabacum, N.carelusesens, O.stavia, P.hybrida cv. Mitchell, Populus sp., P.vittata, Thellungiella sp., T. aestivum, Z.mays, V. vinifera. More details can be found at online website: (https://www.agrisera.com/en/artiklar/v-atpase-epsilon-subunit-o-tonoplast-hatpase.html).

Primary antibody: H+-ATPase; Agrisera; Cat. No. AS07 260; dilution 1:2,000
H+-ATPase was suitable for WB, IF, IL and reacts with [global antibody] di- and moncots, conifers, ferns, mosses, green algae or [cellular compartment marker] plasma membrane. More details can be found at online website: (https://www.agrisera.com/en/artiklar/hatpase-plasma-membrane-hatpase.html).

Eukaryotic cell lines

Policy information about cell lines and Sex and Gender in Research

Cell line source(s)	Yeast cell lines containing BY4741 and fet3fet4 mutant, from Prof. Jian-Feng Ma, Okayama University, Japan.
Authentication	BY4741 can be authenticated by displaying on basal yeast culture medium. fet3fet4 can be authenticated by displaying on basal yeast culture medium with iron supply.
Mycoplasma contamination	The cell lines were not tested for mycoplasma contamination.
Commonly misidentified lines (See ICLAC register)	No commonly misidentified cell lines were used.

Dual use research of concern

Policy information about dual use research of concern

Hazards

Could the accidental, deliberate or reckless misuse of agents or technologies generated in the work, or the application of information presented in the manuscript, pose a threat to:

No	Yes	
☒	☐	Public health
☒	☐	National security
☒	☐	Crops and/or livestock
☒	☐	Ecosystems
☒	☐	Any other significant area

Experiments of concern

Does the work involve any of these experiments of concern:

No	Yes	
☒	☐	Demonstrate how to render a vaccine ineffective
☒	☐	Confer resistance to therapeutically useful antibiotics or antiviral agents
☒	☐	Enhance the virulence of a pathogen or render a nonpathogen virulent
☒	☐	Increase transmissibility of a pathogen
☒	☐	Alter the host range of a pathogen
☒	☐	Enable evasion of diagnostic/detection modalities
☒	☐	Enable the weaponization of a biological agent or toxin
☒	☐	Any other potentially harmful combination of experiments and agents

Plants

Seed stocks	The wild-type soybean seed stocks mentioned in the text are sourced from the seed market and cultivated using laboratory potting methods. The transgenic materials come from transformations conducted in the laboratory and are also cultivated using laboratory potting methods.
Novel plant genotypes	The stable gene knockout mutants were obtained using CRISPR-Cas9 technology in the soybean (Glycine max) genotype Williams 82. The guide RNA sequence for each mutant is shown in Table S1. Transgenic seedlings were then generated through Agrobacterium tumefaciens (EHA105)-mediated transformation. The predicted editing sites in T1 seedlings were sequenced and those with frameshift mutations were selected. T2 homozygous seeds were collected for phenotypic analysis. For all transgenic lines, at least two independent lines were analyzed.
Authentication	The wild type used in this study is commercially available. All targeted mutation sites have been identified through gene sequencing, but we have not further confirmed the presence of secondary effects.
